# Accelerating minimap2 for whole-genome alignment

**DOI:** 10.1093/bioinformatics/btag083

**Published:** 2026-02-19

**Authors:** Ghanshyam Chandra, Md Vasimuddin, Sanchit Misra, Chirag Jain

**Affiliations:** Department of Computational and Data Sciences, Indian Institute of Science, Bangalore, KA 560012, India; Parallel Computing Lab, Intel Corporation, Bangalore, KA 560103, India; Parallel Computing Lab, Intel Corporation, Bangalore, KA 560103, India; Department of Computational and Data Sciences, Indian Institute of Science, Bangalore, KA 560012, India

## Abstract

**Summary:**

Recent advances in long-read sequencing and genome assembly techniques have enabled the generation of high-quality assemblies, often comprising megabase-scale sequences that span entire chromosomes. This results in longer but fewer sequences per genome, which affects the parallelization efficiency of whole-genome alignment tools. Current methods that assign one thread per query sequence now face suboptimal CPU use and longer runtimes because the processing of fewer sequences leaves many threads idle. We present mm2-plus, a fast and efficient method for whole-genome alignment, built upon the commonly used minimap2 aligner. Our improvements include a fine-grained parallel chaining algorithm and a fast method for differentiating primary and secondary chains. These optimizations accelerate the alignment of human, plant, and primate genomes by 1.6× to 7.2× without compromising accuracy.

**Availability and implementation:**

Source code is available at https://github.com/at-cg/mm2-plus and https://doi.org/10.5281/zenodo.18220923.

## 1 Introduction

Improvements in long-read sequencing technologies have enabled routine assembly of high-quality, near-complete genome sequences at affordable costs. Several genome assembly projects, including the Vertebrate Genomes Project (Rhie *et al.* 2021), the Darwin Tree of Life project (Blaxter *et al.* 2022), and the Human Pangenome Reference Consortium (Liao *et al.* 2023), have accelerated the rate at which new assemblies are generated and deposited into public databases. Such efforts also underscore the need for fast algorithms to facilitate comparison of these sequences at scale and gather new insights into evolution and genetic variation.

Whole-genome alignment has been a fundamental problem in bioinformatics for over two decades, ever since the initial reconstruction of the first animal and plant genomes (Bray *et al.* 2003, Brudno *et al.* 2003). Commonly used whole-genome alignment tools use fast algorithms to compute local alignments while accounting for the presence of rearrangements, inversions, transpositions, and duplications. The exact dynamic programming algorithm for computing local alignments requires O(mn) time, where *m* and *n* are the lengths of two sequences (Smith and Waterman 1981). As *m* and *n* approach the lengths of entire genomes, the exact algorithm becomes too time-consuming in practice. Accordingly, modern whole-genome alignment tools use different heuristic techniques (Harris 2007, Kiełbasa *et al.* 2011, Li 2018, Lin and Hsu 2020, Song *et al.* 2022). These heuristic techniques are effective in practice; e.g. the benchmarking of whole-genome aligners in the Alignathon competition (Earl *et al.* 2014) showed that the available methods exhibit good accuracy when aligning genomes over close evolutionary distances.

Over the last few years, the contig lengths of genome assemblies have dramatically increased. Complete or near-complete assemblies of large genomes are now common (Kovaka *et al.* 2023, Liao *et al.* 2023). These advancements motivate the need for whole-genome aligners that scale to large, repetitive genomes. Recent efforts on improving the speed of whole-genome alignment involve optimization of data structures (Marçais *et al.* 2018, Li 2021), the use of cache-aware algorithms (Myers *et al.* 2025), and the use of GPU hardware accelerators (Goenka *et al.* 2020, Gundabolu *et al.* 2021, Gulhan *et al.* 2025).

Whole-genome aligners take a query genome and a reference genome as input. Each genome can be assumed to be a collection of DNA sequences, e.g. contigs or scaffolds, with non-uniform sequence lengths. The common way of implementing multithreading in alignment tools, e.g. in minimap2 (Li 2018, 2021) and Mummer4 (Marçais *et al.* 2018), is to distribute the query sequences across the CPU threads. In this approach, each thread aligns the query sequences assigned to it to the entire reference genome. This approach is simple and efficient for aligning a fragmented genome assembly comprising thousands of contigs or for aligning a large set of reads. However, this approach has poor efficiency if the query genome contains a small number of long sequences. For instance, in telomere-to-telomere (T2T) complete genomes containing one sequence per chromosome (Nurk *et al.* 2022), many threads remain idle, leading to poor workload balance. While using minimap2 to align high-quality genome assemblies of various species, we find an average CPU utilization of <7% on a 48-core processor, indicating a significant gap between the actual performance and the theoretical peak performance.

**Figure 1:**
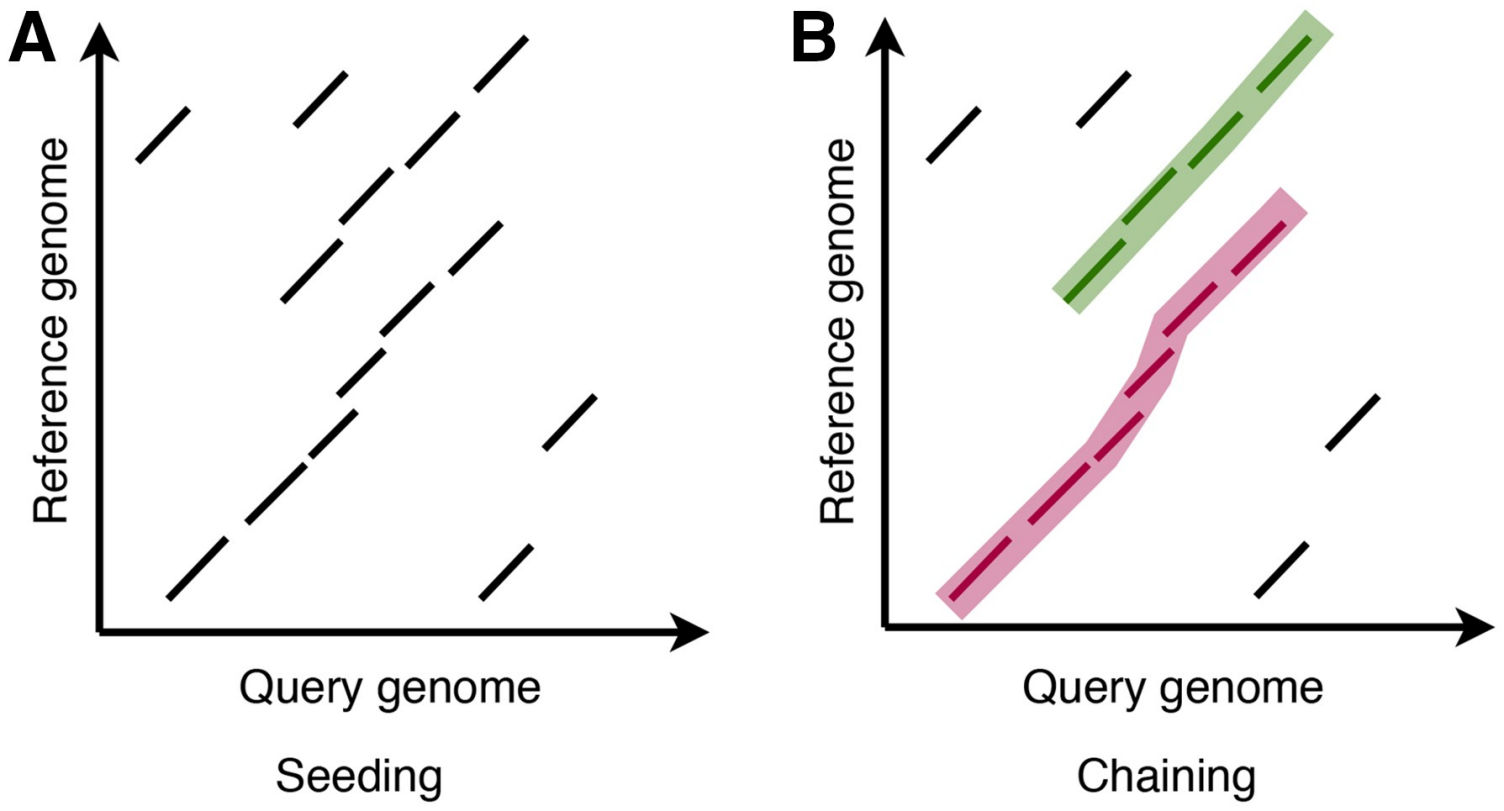
Illustration of the seeding and chaining steps. (A) Each black diagonal line segment illustrates an exact *k*-mer match (anchor) between two genomes. (B) Computation of long chains comprising multiple anchors. In this example, there are two chains. The two chains have overlapping intervals on the query genome. The chain highlighted in magenta color has a higher score due to a greater number of anchors. Accordingly, the magenta chain is considered as primary, whereas the green chain is considered as secondary.

In this work, we propose optimizations to accelerate whole-genome alignment on multicore processors. Our optimizations are applicable to any genome alignment tool that follows the seed-chain-extend heuristic method (Brudno *et al.* 2003, Kent *et al.* 2003, Li 2018, Marçais *et al.* 2018). Seed-chain-extend is a widely used heuristic method that involves (i) computing short exact matches between two genomes called *anchors*, (ii) combining groups of anchors into longer, high-scoring *chains* (Fig. 1), (iii) classifying a chain as either *primary* or *secondary* when the same region of the query genome aligns to two regions in the reference, and (iv) computing base-to-base alignments by extending the anchors of each chain.

We profiled the percentage contribution of each step in minimap2 toward the total whole-genome alignment runtime (Figs S1 and S2, available as supplementary data at *Bioinformatics* online). The profiling results show that all four steps of the seed-chain-extend workflow contribute significantly to the overall runtime. Therefore, we designed multiple optimizations to accelerate these steps. First, we developed a fine-grained parallel algorithm for chaining. Our algorithm enables parallel chaining of a single query sequence across multiple threads. Second, we implemented a fast interval tree-based algorithm for classifying chains as primary or secondary. Third, we replaced sequential sorting routines with parallel sorting to accelerate the seeding stage. Fourth, we optimized the extension stage by using a SIMD (single instruction multiple data)-parallel alignment library (Kalikar *et al.* 2022). Together, these optimizations resulted in 1.6−7.2× speedup for whole-genome alignment while preserving the output accuracy. For example, the original minimap2 implementation requires ∼12 hours of wall-clock time to align the barley genome assembly to the barley reference genome using 48 threads, whereas our implementation, mm2-plus, takes <2 hours.

Besides minimap2, there are other genome aligners available, such as LastZ (Harris 2007), AnchorWave (Song *et al.* 2022), NUCmer (Marçais *et al.* 2018), and FastGA (Myers *et al.* 2025). Although minimap2 is not the most sensitive alignment tool among them, it offers a practical tradeoff between speed and sensitivity (Saada *et al.* 2024, Myers *et al.* 2025). minimap2 is also integrated within assembly-based variant calling methods (Li *et al.* 2018, Ebert *et al.* 2021, Heller and Vingron 2021). For these reasons, we chose minimap2 as the baseline for implementing and evaluating our optimizations.

## 2 Materials and methods

We developed mm2-plus (https://github.com/at-cg/mm2-plus) over the minimap2 v2.30 code base (Li 2018). Both mm2-plus and minimap2 accept the query and reference genomes in the standard FASTA format and output the computed alignments in either PAF (https://github.com/lh3/miniasm/blob/master/PAF.md) or SAM (https://samtools.github.io/hts-specs/SAMv1.pdf) formats.

In minimap2, the sequences in the query genome are processed in batches. The query sequences within a batch are processed independently in parallel by utilizing only one thread per sequence. Processing a query genome in batches helps in avoiding excessive memory usage (Table S1, available as supplementary data at *Bioinformatics* online). However, a batch may not contain enough number of query sequences to utilize all the available CPU threads efficiently. In the following subsections, we present the details of mm2-plus optimizations.

### 2.1 Parallel algorithm for chaining

The input to the chaining algorithm is a set of anchors (exact *k*-mer matches) between query sequences and the reference genome. The output of this step is a set of chains, where each chain comprises multiple anchors that can be joined to form an alignment (Fig. 1).

Chaining of anchors is performed using a well-established dynamic programming algorithm that requires O(N log N) time, where *N* denotes the number of anchors between a query sequence and the reference genome. We refer the readers to (Abouelhoda and Ohlebusch 2005, Li 2018, Jain *et al.* 2022) for the chaining problem formulation and details of the dynamic programming algorithm. Minimap2 uses a range tree-based implementation for chaining (Li 2021). A range tree data structure enables efficient search over the anchor scores. Our profiling results indicate that the chaining algorithm consumes 38%–56% of the overall time in minimap2 (Fig. S2, available as supplementary data at *Bioinformatics* online), highlighting the need for a more efficient approach.

In mm2-plus, in addition to parallelizing across the query sequences in a batch, we also parallelize the chaining of each query sequence using multiple threads to achieve better CPU utilization and runtime. Note that each anchor is denoted by its (i) position on the query genome, (ii) position on the reference genome, and (iii) a binary value indicating whether the match occurred on the forward or the reverse strand of the reference sequence. Our approach to parallelizing the chaining algorithm is partly inspired by the parallel chaining algorithm of Abouelhoda and Mohamed (2010).

In an array of anchors where the anchors are sorted by their positions on the reference genome, there will likely exist several anchor pairs that cannot belong to the same chain. The chaining scores of these anchors are independent and can be computed in parallel. We exploit the property that a pair of anchors between a query sequence and two different sequences of the reference genome cannot be chained together. The number of such anchor pairs is large in practice due to repeats. Thus, given a set of anchors from a single query sequence, we partition the anchors according to the reference genome sequence they match with. Accordingly, the number of partitions is twice the number of sequences in the reference genome (considering both the forward and reverse orientations). Importantly, this approach does not alter the chaining output. An illustration of this partitioning approach is available in Fig. S3, available as supplementary data at *Bioinformatics* online. We empirically checked the distribution of partition sizes and concluded that this approach should offer a good amount of parallelism to accelerate chaining (Fig. S4, available as supplementary data at *Bioinformatics* online). Subsequently, we chain each partition independently using the default chaining algorithm of minimap2. This way of workload decomposition for parallel processing differs from the approaches discussed by Abouelhoda and Mohamed (2010). Their approaches are applicable to chaining between a pair of sequences and do not exploit the fact that there are multiple sequences in a reference genome.

We assign the anchor partitions to CPU threads carefully because some partitions may be bigger than others. We use dynamic assignment of the threads to each partition to handle the non-uniform distribution of anchors across reference sequences. We use the above parallelization technique to accelerate both the recursion and traceback phases of the dynamic programming. Compared to more sophisticated parallelization approaches, the proposed approach does not involve synchronization overheads because different threads can work independently on each partition.

### 2.2 Faster algorithm for marking primary chains

The output of the chaining algorithm is an array of anchor chains, denoted as C. The array C is ordered in descending order of the chain scores. Suppose the number of chains in C is *n*. The distinction between primary and secondary chains is required in minimap2 when a segment of a query genome aligns to two or more positions in the reference genome (Fig. 1).

One can identify the start and end coordinates of a chain over the query genome using the query start coordinate of the first anchor and the query end coordinate of the last anchor in the chain, respectively. For a chain *c*, let us denote *c*.*s* and *c*.*e* as its query start and query end coordinates, respectively. If there exist two or more chains having significant overlaps with each other in the query sequence, then the chain with the highest score is considered primary in minimap2, and the others are considered secondary. Before discussing our interval tree-based implementation, we review the quadratic-time algorithm used in minimap2, which contributes up to 35% to the total runtime (Fig. S2, available as supplementary data at *Bioinformatics* online).

For two chains $c_{i}$ and $c_{j}$, their *overlap fraction* is defined as the ratio of their overlap length and the length of the shorter chain. Mathematically, we can write this as the ratio of $\max\left(0,\min(c_{i}.e,c_{j}.e)-\max(c_{i}.s,c_{j}.s)\right)$ to $\min(c_{i}.e-c_{i}.s,\;c_{j}.e-c_{j}.s)$. The algorithm used in minimap2 works as follows. Denote the set of primary chains as *Q*. Set *Q* is initially empty. The first chain from C, i.e. the highest-scoring chain, is added to *Q*. Subsequently, the remaining chains are in C[2..n] are considered sequentially. A chain C[i] is marked as secondary if there exists a chain in *Q* that has an overlap fraction above 0.5 with C[i]. Otherwise, C[i] is marked as primary and added to set *Q*. Each iteration of the algorithm linearly scans set *Q*. Accordingly, this algorithm requires $O(n^{2})$ time in the worst case because |Q| can be as large as *n*.

In mm2-plus, we utilize an interval tree to avoid linear scans of *Q*. An interval tree is a standard data structure for storing and querying intervals. An interval tree comprising *m* intervals supports (i) addition of a new interval in O(log m) time and (ii) querying of all intervals that intersect with any given interval in O(log m+k) time, where *k* denotes the number of intervals produced by the query (de Berg *et al.* 2008). We use the same approach as minimap2 but maintain the intervals associated with the chains of set *Q* in an interval tree. While processing the chain C[i], we retrieve all the overlapping intervals in *Q* by making a query in the tree. As a result, each iteration now takes O(log |Q|+k) time. In practice, k≪n. Accordingly, the proposed algorithm uses O(n log n) time in practice.

### 2.3 Other optimizations

We implemented two additional optimizations in mm2-plus: (i) the AVX (advanced vector extensions)-based parallel base-to-base alignment code from mm2-fast (Kalikar *et al.* 2022) and (ii) the use of a parallel sorting algorithm (Carlini *et al.* 2023), replacing the sequential sorting algorithm in minimap2. The sorting algorithms are used for various purposes in minimap2, such as ordering anchors by their starting positions on the reference genome prior to chaining.

## 3 Results

### 3.1 Experimental setup

We used the following datasets in our evaluation: (i) Barley–Barley, (ii) Maize–Maize, (iii) Human–Bonobo, and (iv) Human–Human. For a dataset named X–Y, we align a genome of species X to a genome of species Y. The contiguity of these genomes is consistent with the quality of the latest genome assemblies. For example, the N50 scaffold lengths in all genomes exceed 100 Mbp (Table S2, available as supplementary data at *Bioinformatics* online). The N50 statistic implies that half of the genome bases are represented in sequences of this length or greater. One of these datasets (Human–Bonobo) corresponds to an inter-species genome comparison. The remaining are intraspecies genome comparisons. We used the default alignment parameters from the minimap2 documentation. To maintain reproducibility, the commands used for running all software are available in Table S3, available as supplementary data at *Bioinformatics* online. We conducted our experiments on four different CPU architectures: Intel^®^ Xeon^®^ Platinum 8592+ (Emerald Rapids), Intel^®^ Xeon^®^ Platinum 8480 L (Sapphire Rapids), Intel^®^ Xeon^®^ Platinum 6248R (Cascade Lake), and AMD EPYC™ 7763 (Milan). The hardware specifications of these systems are available in Table S4, available as supplementary data at *Bioinformatics* online.

### 3.2 Performance evaluation

**Figure 2:**
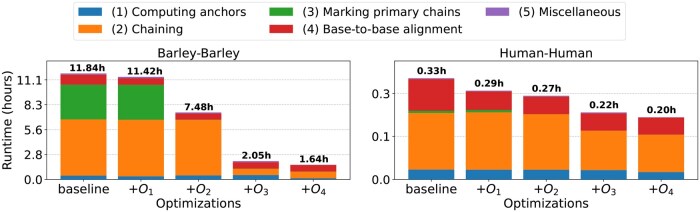
End-to-end runtime using minimap2 (baseline) and the different versions of mm2-plus. We progressively enabled the four optimizations $O_{1}$, $O_{2}$, $O_{3}$, and $O_{4}$ in mm2-plus for a detailed evaluation. The labeling of the four optimizations is done as $O_{1}$ [faster base-to-base alignment using AVX (Kalikar *et al*. 2022)], $O_{2}$ (faster algorithm for marking primary chains), $O_{3}$ (parallel chaining), and $O_{4}$ (parallel sorting). The y-axes indicate wall-clock time. In each bar, we show the runtime contribution of different steps using different colors. We conducted this experiment using 48 threads.

We compared the runtime of mm2-plus with minimap2 using the four datasets. We measured the impact of each optimization, labeled as $O_{1}$ [faster base-to-base alignment using AVX (Kalikar *et al.* 2022)], $O_{2}$ (faster algorithm for marking primary chains), $O_{3}$ (parallel chaining), and $O_{4}$ (parallel sorting). We compared minimap2 with different versions of mm2-plus in which we enabled the optimizations progressively. In other words, we compared minimap2, mm2-plus ($O_{1}$ enabled), mm2-plus ($O_{1}+O_{2}$ enabled), mm2-plus ($O_{1}+O_{2}+O_{3}$ enabled), and mm2-plus ($O_{1}+O_{2}+O_{3}+O_{4}$ enabled). This experiment is useful to evaluate the advantages of each optimization as well as measure their combined effect. We conducted this experiment using 48 threads on a Cascade Lake CPU. We present the runtime results obtained using the Barley–Barley and Human–Human datasets in Fig. 2. Runtimes using the other two datasets are shown in Fig. S5, available as supplementary data at *Bioinformatics* online. We also show a comparison of runtimes and memory usage of minimap2, mm2-plus, and mm2-fast (Kalikar *et al.* 2022) in Figs S6 and S7, available as supplementary data at *Bioinformatics* online, respectively.

We draw the following conclusions from the results. When all optimizations are enabled, mm2-plus is 1.6−7.2× faster than minimap2. We also conclude that mm2-plus is 1.3−6.2× faster than mm2-fast (Kalikar *et al.* 2022). Compared to minimap2, the CPU utilization improves from 2.3% to 14.2% using Barley from 2.3% to 14.2% using Barley–Barley dataset, from 4.7% to 18.2% using the Maize–Maize dataset, from 6.6% to 10.9% using the Human–Bonobo dataset, and from 5.8% to 9.2% using the Human–Human dataset–Barley dataset, from 4.7% to 18.2% using the Maize–Maize dataset, from 6.6% to 10.9% using the Human–Bonobo dataset, and from 5.8% to 9.2% using the Human–Human dataset (Fig. S12, available as supplementary data at *Bioinformatics* online).

Even with the proposed optimizations, the CPU utilization using mm2-plus still remains well below the ideal 100%. To investigate the sources of this inefficiency, we performed additional experiments to quantify the contributing overheads. Our experiments indicate that the sequential portions in the overall workflow and the load imbalance due to non-uniform sequence lengths in every batch are the primary factors that affect CPU utilization (Figs S13 and S14, available as supplementary data at *Bioinformatics* online).

Our speedups are much better using plant genomes. This is because these genomes have abundant repeat elements, which are advantageous for our algorithm. Our optimizations $O_{2}$ and $O_{3}$ work better on barley and maize plant genomes because (i) alignment of these genomes generates millions of chains (Fig. S8, available as supplementary data at *Bioinformatics* online), making the interval tree-based approach for marking primary chains more efficient and (ii) our parallel chaining algorithm, which leverages anchor partitioning, performs better since query sequence anchors are often distributed across multiple sequences of the reference genome, leading to improved thread utilization. Overall, the results highlight that all the optimizations introduced in mm2-plus are practically useful and contribute to reducing runtime across all datasets.

Our optimizations in mm2-plus remain compatible with the range of Intel and AMD CPUs. This is demonstrated by our benchmarking of mm2-plus using Intel and AMD CPU architectures, which shows similar speedups (Fig. S9, available as supplementary data at *Bioinformatics* online). We also evaluated the performance of mm2-plus using <48 threads to assess its efficiency on low-end servers with limited cores (Fig. S10, available as supplementary data at *Bioinformatics* online). The results indicate that mm2-plus remains faster than minimap2 using fewer threads. The highest speedup is achieved using ≥16 threads. Beyond 16 threads, the runtimes of mm2-plus and minimap2 plateau across all datasets.

There also exist accelerated versions of minimap2 for GPUs, e.g. mm2-ax (Sadasivan *et al.* 2023) and its successor tool mm2-gb (Dong *et al.* 2024). We evaluated the runtime of mm2-gb on our datasets using an NVIDIA A100 GPU and observed that it was slower than minimap2 executed on a 48-thread Cascade Lake CPU (Table S8, available as supplementary data at *Bioinformatics* online).

Finally, we ensured that the optimizations in mm2-plus do not affect the quality of alignments. To validate this, we checked the accuracy of alignments by variant calling. For each dataset (i.e. Barley–Barley, Maize–Maize, Human–Human, and Human–Bonobo), we used the alignment between the query genome and the reference genome to call variants using paftools (https://github.com/lh3/minimap2/blob/master/misc/paftools.js). We considered the set of variants obtained using minimap2 alignments as the ground truth. We observed at most a 0.0097% drop in the F1 score across all datasets (Table S5, available as supplementary data at *Bioinformatics* online). These differences in the mm2-plus output are introduced by subtle changes in our implementation; e.g. the sorting algorithm in minimap2 is not stable, whereas the sorting algorithm in mm2-plus is stable. We also verified that (i) the fraction of the query genome aligned and (ii) the fraction of the reference genome aligned using minimap2 and mm2-plus are identical across all datasets (Table S6, available as supplementary data at *Bioinformatics* online).

All assemblies used in our benchmark exhibit high contiguity. We carried out a separate experiment to assess the advantage of mm2-plus on fragmented long-read assemblies. In such cases, minimap2 is expected to achieve better CPU utilization compared to complete assemblies because threads can process contigs (or scaffolds) in parallel. We compared minimap2 and mm2-plus using a phased assembly of *L. vulgaris* (common newt) species from the Darwin Tree of Life Project (Blaxter *et al.* 2022). The reference genome (GCA_964263255.1) has a total size of 24.2 Gbp, 15265 scaffolds, and a scaffold N50 of 1.9 Gbp. The query genome (GCA_964261385.1) has a total size of 20.8 Gbp, 191290 scaffolds, and a scaffold N50 of 591 kbp. Using mm2-plus on this dataset, we observed 1.5× speedup over minimap2, which, as anticipated, is lower than the speedups observed for the other datasets. Nonetheless, *de novo* assembly methods and sequencing technologies continue to advance rapidly and are likely to further improve the quality of assemblies produced in various projects.

## 4 Discussion

Modern genome assemblies comprise longer and fewer sequences when compared to the assemblies generated from short reads or noisy long reads. In this work, we highlighted that the common approach for parallelizing genome alignment on multi-core processors is inefficient. The parallelism available at the level of query sequences is limited, raising a need for an alternative approach. We exclusively focused on improving one of the widely used genome aligners, minimap2 (Li 2018). Our profiling experiments using minimap2 revealed that the CPU utilization is consistently low across multiple datasets. To address this limitation, we developed mm2-plus, which improves the performance of the most time-consuming components of minimap2.

We implemented optimizations in mm2-plus that helped us achieve faster runtime without affecting the quality of alignments. These included faster algorithms for chaining, marking of primary chains, sorting, and base-to-base alignment. Collectively, these optimizations resulted in 1.6−7.2× speedup for whole-genome alignment across a diverse set of datasets.

There remain opportunities for further improvement. CPU utilization in mm2-plus remains well below the theoretical maximum due to various sources of overhead (Figs S13 and S14, available as supplementary data at *Bioinformatics* online). Additional performance gains could be achieved by (i) developing novel fine-grained, work-optimal parallel algorithms for the seeding, chaining, and extension stages, (ii) designing memory-efficient seeding and chaining methods to enable larger batch sizes and increased parallelism, and (iii) employing dynamic scheduling strategies to mitigate load imbalance arising from uneven sequence lengths.

We note that minimap2 (and, by extension, mm2-plus) can reliably compute whole-genome alignments when sequence divergence is ∼5% or less (Myers *et al.* 2025). For more highly diverged species, the accuracy of both methods declines. This loss of sensitivity at higher divergence arises from the filtering of *k*-mer matches during the chaining stage.

This work complements our earlier work on accelerating long-read mapping (Kalikar *et al.* 2022). To make it convenient for users, we have ported the optimizations from (Kalikar *et al.* 2022) into mm2-plus. Therefore, mm2-plus can be utilized not only for whole-genome alignment but also for fast long-read mapping and all-vs-all read alignment (Fig. S11, available as supplementary data at *Bioinformatics* online). Therefore, in any genomics workflow where alignment tasks are a bottleneck, users may benefit from using mm2-plus.

## Supplementary Material

btag083_Supplementary_Data

## Data Availability

The source code of mm2-plus is available at https://github.com/at-cg/mm2-plus and https://doi.org/10.5281/zenodo.18220923. All our experiments were done using publicly available data (Table S2, available as supplementary data at *Bioinformatics* online).
