# Supplementary material for: Accelerating minimap2 for whole-genome alignment
Source: Bioinformatics. 2026 Feb 19;42(3):btag083. doi: 10.1093/bioinformatics/btag083 (PMC12980334; doi:10.1093/bioinformatics/btag083)
Supplement: btag083_Supplementary_Data [file btag083_supplementary_data.pdf]

Accelerating minimap2 for whole-genome alignment - Supplementary Data

Ghanshyam Chandra, Md. Vasimuddin, Sanchit Misra and Chirag Jain

---

Contents

List of Figures

S1 Illustration of the main computational steps in minimap2 and the proposed optimizations. . . 2
S2 Pie charts to visualize the runtime profiling results of minimap2. 2
S3 An illustration of the anchor partitioning algorithm for parallel chaining. 3
S4 Illustration of the distribution of the count of anchors over the sequences of reference genome. 3
S5 End-to-end runtime (wall-clock time) using minimap2 (baseline) and the different versions of mm2-plus. . 4
S6 Runtime comparison of minimap2, mm2-fast, and mm2-plus for whole-genome alignment. . 4
S7 Memory usage comparison between minimap2, mm2-fast, and mm2-plus. 5
S8 Illustration of the count of chains computed between each sequence of the query genome and the reference genome across various datasets. . 5
S9 Speedups of mm2-plus on various CPU architectures. 6
S10 Comparison of mm2-plus and minimap2 runtimes using different number of threads. 6
S11 Comparison of runtimes of minimap2, mm2-fast, and mm2-plus for long-read mapping and all-vs-all read alignment use-cases. . 7
S12 Profiling CPU utilization of mm2-plus and minimap2. 8
S13 Illustration of the overheads that affect CPU utilization of mm2-plus. 9
S14 Evaluation of various overheads in mm2-plus during parallel execution. 9

List of Tables

S1 Effect of increasing batch size on runtime and memory usage. 10
S2 Details of the datasets used for benchmarking. 10
S3 Commands used for running the experiments. 11
S4 Overview of the architectural details for the four processors used in the experiments. 12
S5 Comparison of the variant calls obtained using mm2-plus and minimap2 alignments. 12
S6 Fraction of query and reference genome aligned using minimap2 and mm2-plus. 12
S7 Details of the long-read datasets used in our benchmark. 13
S8 Runtime and memory-usage comparison of mm2-gb and minimap2. 13

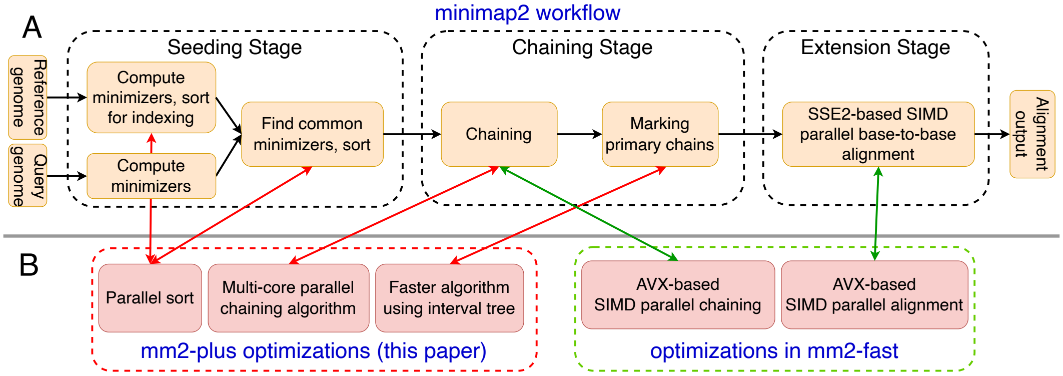

Figure S1: **Illustration of the main computational steps in minimap2 and the proposed optimizations**. **(A)** The key steps involved in the seed-chain-extend alignment workflow of minimap2. **(B)** The proposed techniques implemented in mm2-plus to accelerate the individual steps. We also used optimizations from our previous work, mm2-fast, as shown above.

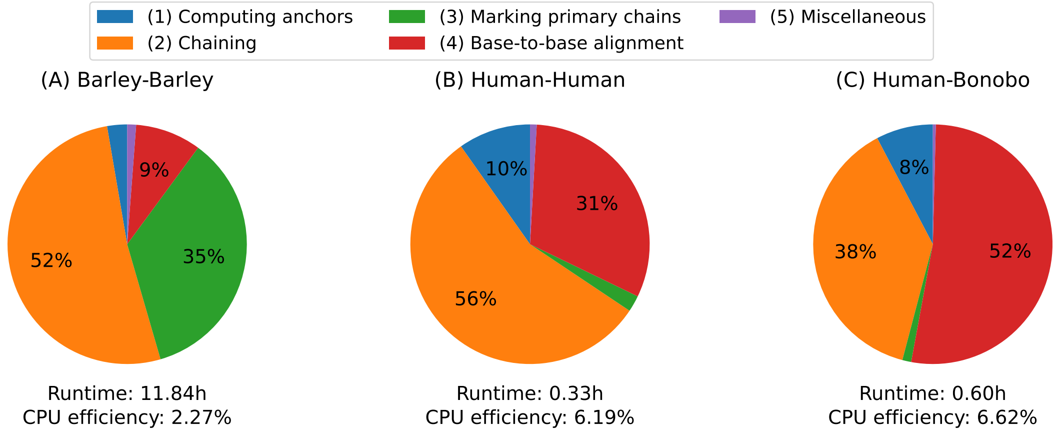

Figure S2: **Pie charts to visualize the runtime profiling results of minimap2**. We profiled the runtime contribution of various steps in the seed-chain-extend workflow of minimap2 to identify the time-consuming steps. We conducted this experiment using three datasets: (a) Barley-Barley, (b) Human-Human, and (c) Human-Bonobo. Minimap2 has four key steps: (1) Seeding step for *computing anchors*, (2) Anchor *chaining*, (3) *Marking primary chains*, and (4) Extension step, i.e., *base-to-base alignment*. In the profile, the remaining time apart from these four steps is presented as *miscellaneous*. We conducted this experiment using 48 threads of a Cascade Lake CPU. The results suggest that the algorithms used in all four steps must be improved to achieve a significant reduction in the overall runtime. Another important observation is that the CPU utilization ranges from 2.27% to 6.62%, suggesting that most processor cores predominantly remain idle during the program execution due to poor load balance among threads. Total runtime (i.e., wall clock time) varies from 11.84 hours (Barley-Barley) to 0.33 hours (Human-Human). Barley genome comparisons require significantly more time because barley genomes are longer and more repetitive compared to human genomes.

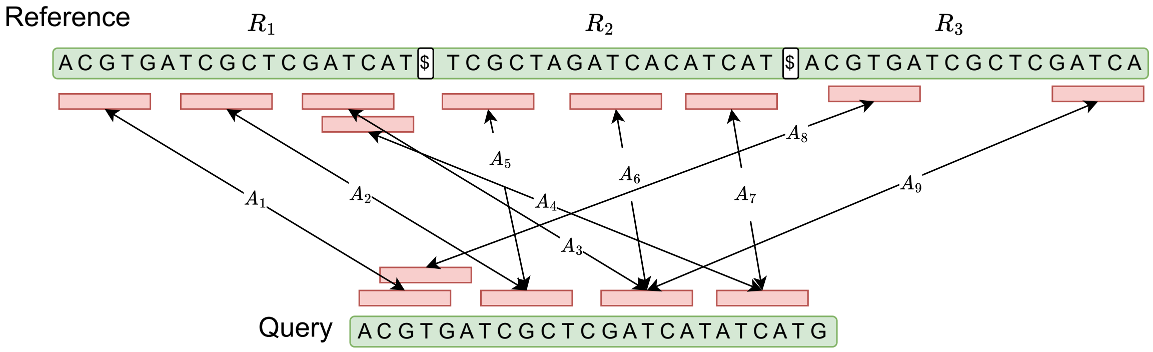

Figure S3: **An illustration of the anchor partitioning algorithm for parallel chaining.** In the above example, the reference genome contains three sequences labeled as R_1, R_2, R_3. They are concatenated together using a '$' delimiter in our implementation. We ignore the reverse complement of these sequences to keep this illustration simple. We have nine anchors in total from the query sequence. These anchors would be split into the following three partitions based on the reference sequence they match with: $\{A_1, A_2, A_3, A_4\}$, $\{A_5, A_6, A_7\}$, and $\{A_8, A_9\}$. Subsequently, the chaining algorithm is run independently on each partition.

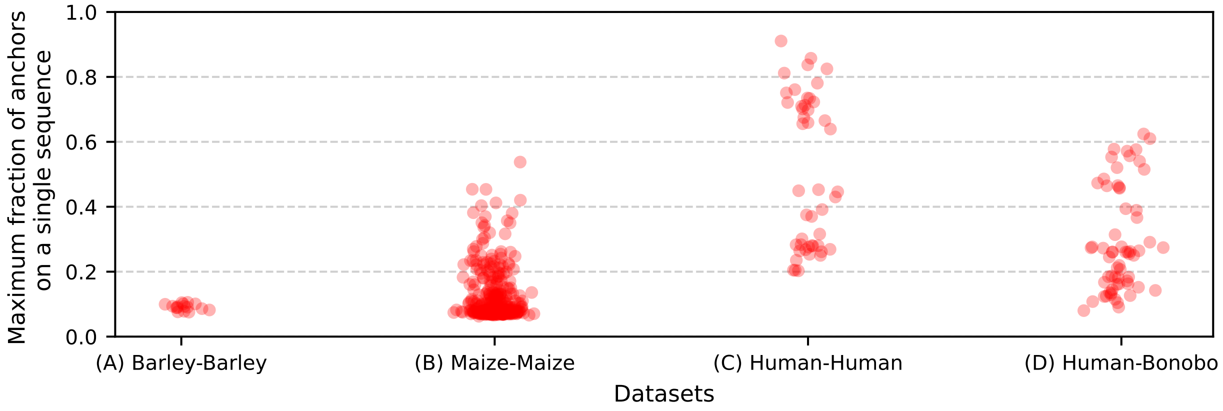

Figure S4: **Illustration of the distribution of the count of anchors over the sequences of reference genome.** For each query sequence (indicated as a red circle), we calculated the fraction of the maximum number of anchors on a single reference sequence relative to the total number of anchors. A higher fraction value indicates that most anchors are located on a single reference sequence; that is, the distribution of anchors is skewed. A lower value indicates that the anchors are scattered across various reference sequences; that is, the distribution is more uniform. We observe lower fraction values in plant genomes than in animal genomes due to the higher presence of inter-chromosomal repeats and mobile elements.

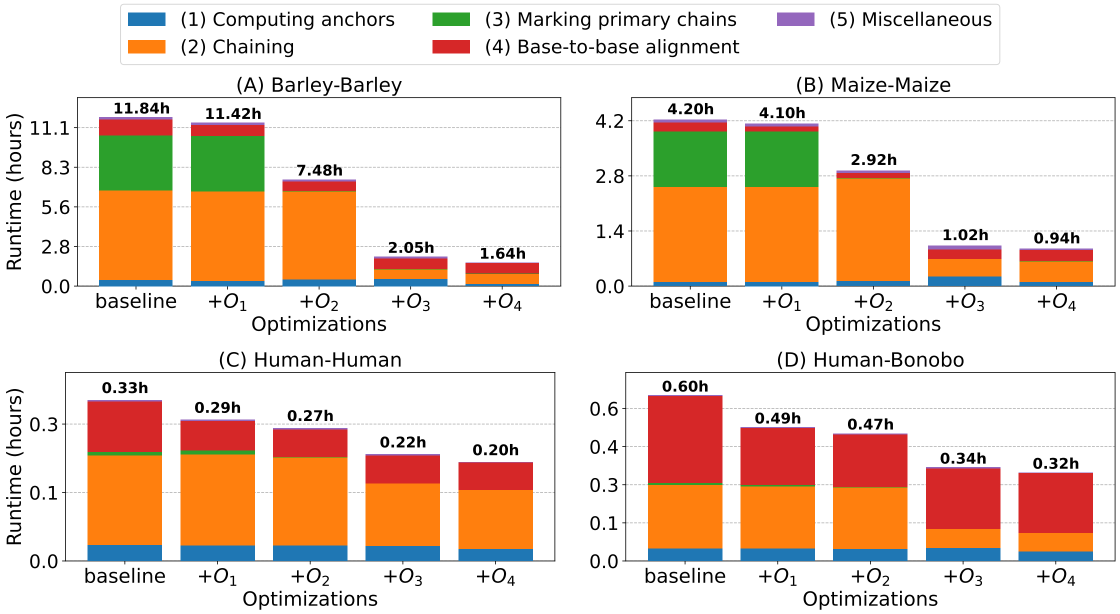

Figure S5: **End-to-end runtime (wall-clock time) using minimap2 (baseline) and the different versions of mm2-plus**. We progressively enabled the four optimizations O_1, O_2, O_3, and O_4 in mm2-plus for a detailed evaluation. The labeling of the four optimizations is done as O_1 (faster base-to-base alignment using AVX), O_2 (faster algorithm for marking primary chains), O_3 (parallel chaining), and O_4 (parallel sorting). In each bar, we show the runtime contribution of different steps using different colors. We conducted this experiment using 48 threads.

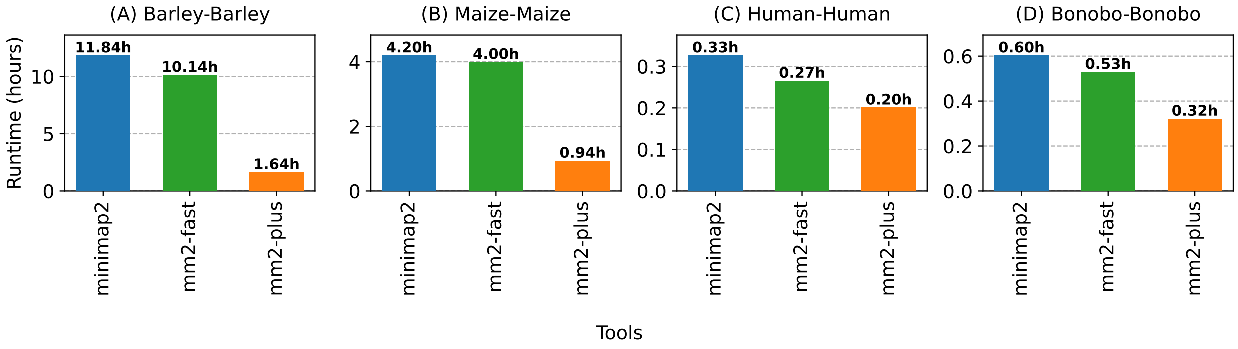

Figure S6: **Runtime comparison of minimap2, mm2-fast, and mm2-plus for whole-genome alignment**. Panels (A)–(D) show runtimes across various datasets. All experiments were conducted using 48 threads on a Cascade Lake CPU.

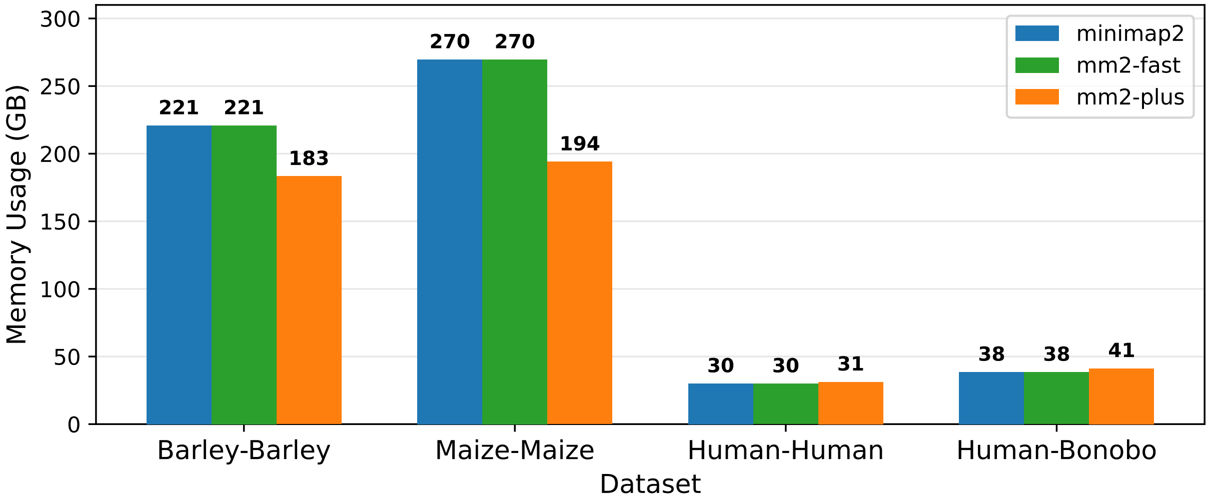

Figure S7: **Memory usage comparison between minimap2, mm2-fast, and mm2-plus**. We conducted this experiment using 48 threads on a Cascade Lake CPU, with all optimizations in `mm2-plus` enabled. We see a lower memory usage using `mm2-plus` on Barley-Barley and Maize-Maize datasets because we used a different memory allocator called `jemalloc` (`https://jemalloc.net`). `jemalloc` employs multi-threaded arenas to eliminate lock contention and size-segregated bins for efficient small-object allocation. It can reduce memory fragmentation and the overall memory usage.

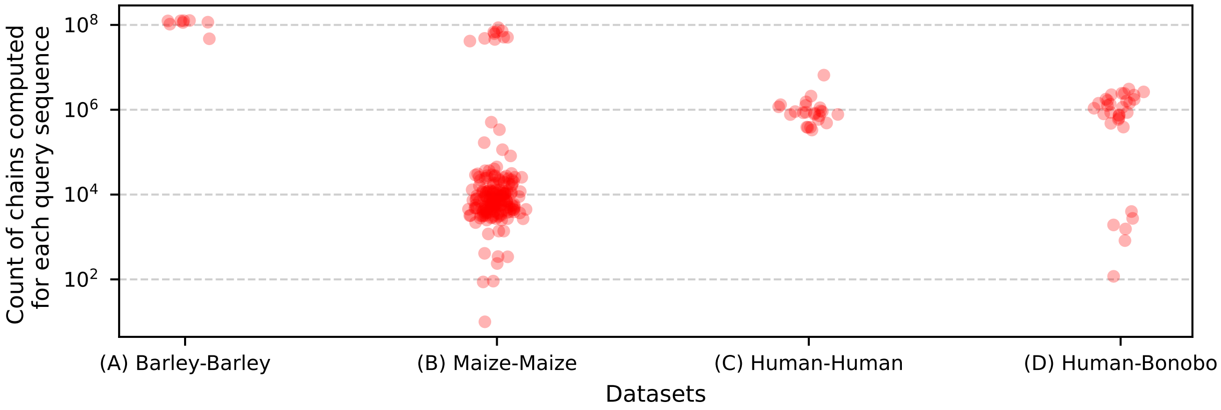

Figure S8: **Illustration of the count of chains computed between each sequence of the query genome and the reference genome across various datasets.** Each red circle represents a query sequence. These chains are further processed and classified as either primary or secondary in a subsequent step of the alignment workflow.

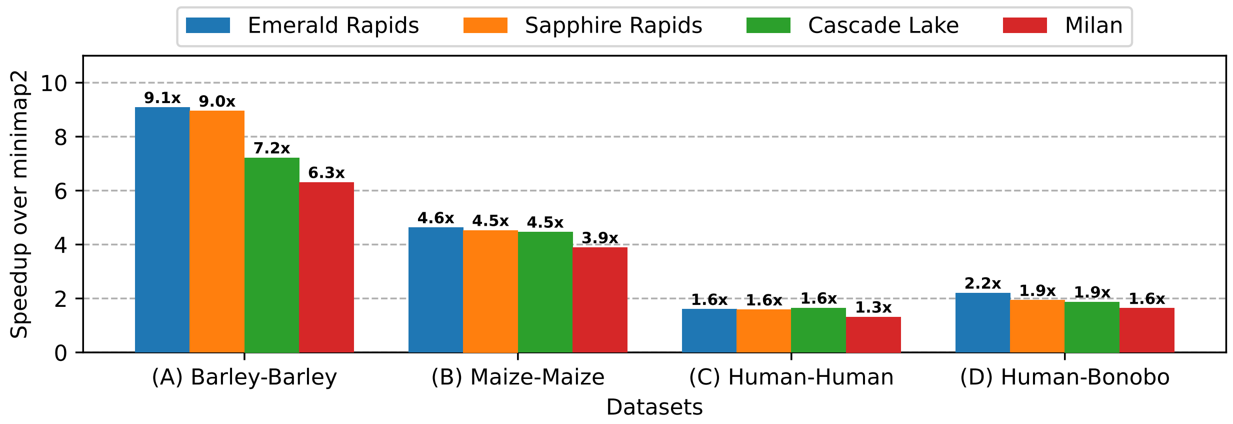

Figure S9: **Speedups of mm2-plus on various CPU architectures.** Our optimizations in mm2-plus are compatible with a range of Intel and AMD CPUs. To demonstrate this, we benchmarked mm2-plus against minimap2 on four different architectures: Intel Emerald Rapids, Intel Sapphire Rapids, Intel Cascade Lake, and AMD Milan, all on single-socket. These processors differ in core counts, SIMD register widths, clock frequencies, cache sizes, etc. (Table S4). We observed a higher speedup on plant genomes than on primate genomes. While our parallel chaining, parallel sorting, and primary-chain marking optimizations are hardware-agnostic, the performance of SIMD-based base-to-base alignment is influenced by the SIMD register width. Specifically, Intel CPUs support AVX2 (256-bit) and AVX512 (512-bit) instructions, whereas AMD CPUs only support AVX2. The figure shows that for Intel processors, the speedup numbers improved with the newer generations of architecture, from Cascade Lake to Emerald Rapids.

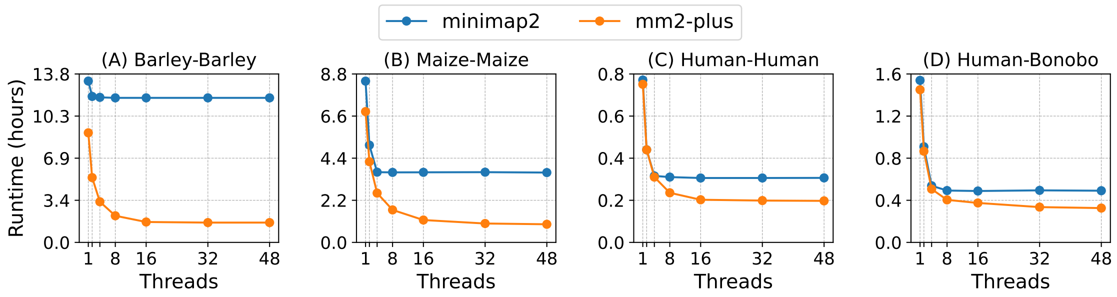

Figure S10: **Comparison of mm2-plus and minimap2 runtimes using different number of threads.**

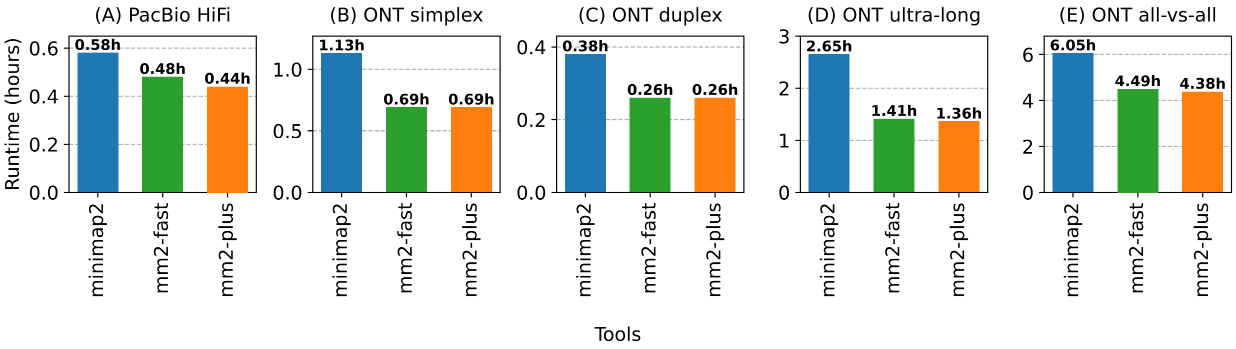

Figure S11: **Comparison of runtimes of minimap2, mm2-fast, and mm2-plus for long-read mapping and all-vs-all read alignment use-cases.** Minimap2 supports a range of alignment tasks, from long-read mapping for genome resequencing to all-vs-all read alignments for *de novo* assembly. To speed up these applications, we ported the optimizations from mm2-fast (including SIMD-parallel chaining and base-to-base alignment) to mm2-plus and added dynamic load balancing to account for non-uniform long read lengths. We benchmarked all three tools using PacBio HiFi and multiple Oxford Nanopore datasets from the HG002 human genome (Table S7) using 48 threads of a Cascade Lake CPU. Panels (A) to (D) show the speedups achieved for long-read mapping to CHM13 human genome (GCA_009914755.4). Panel (E) shows the speedup for all-vs-all alignment of ONT simplex reads. Overall, mm2-plus delivers a speedup over minimap2 ranging from 1.32× to 1.95×, with occasional small gains over mm2-fast due to dynamic load balancing. The commands used to run the experiments are listed in the Table S3. We verified that the long-read alignment output from mm2-plus was identical to minimap2 in all these experiments. See our documentation on GitHub(`https://github.com/at-cg/mm2-plus?tab=readme-ov-file#accuracy-evaluation`) for more details.

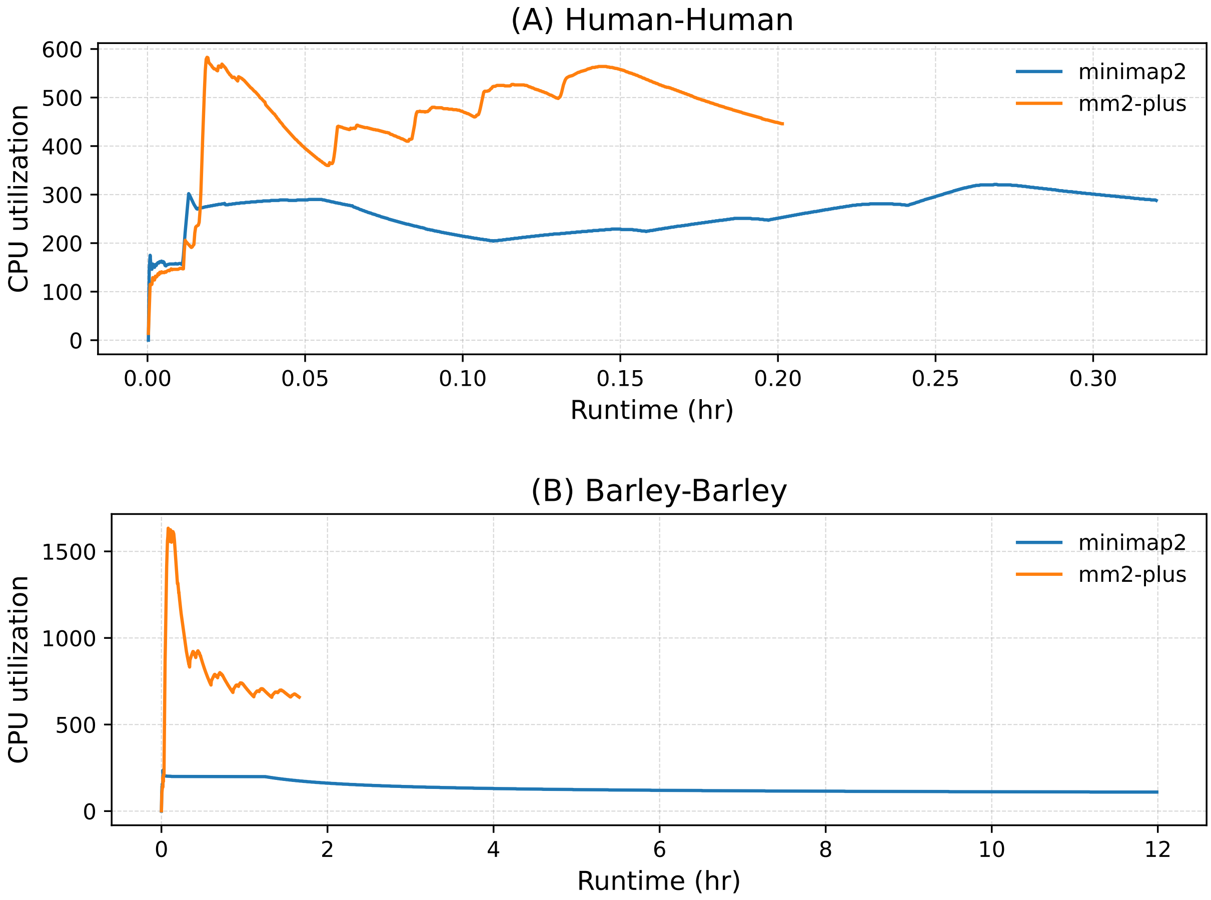

Figure S12: **Profiling CPU utilization of mm2-plus and minimap2 using Human-Human and Barley-Barley datasets.** We conducted this experiment using 48 threads of Cascade Lake CPU. Despite our optimizations in mm2-plus, maximum CPU utilization remains below $\approx 583\%$ for Human-Human dataset and below $\approx 1634\%$ for Barley-Barley dataset. The theoretical maximum utilization with 48 threads is 4800%.

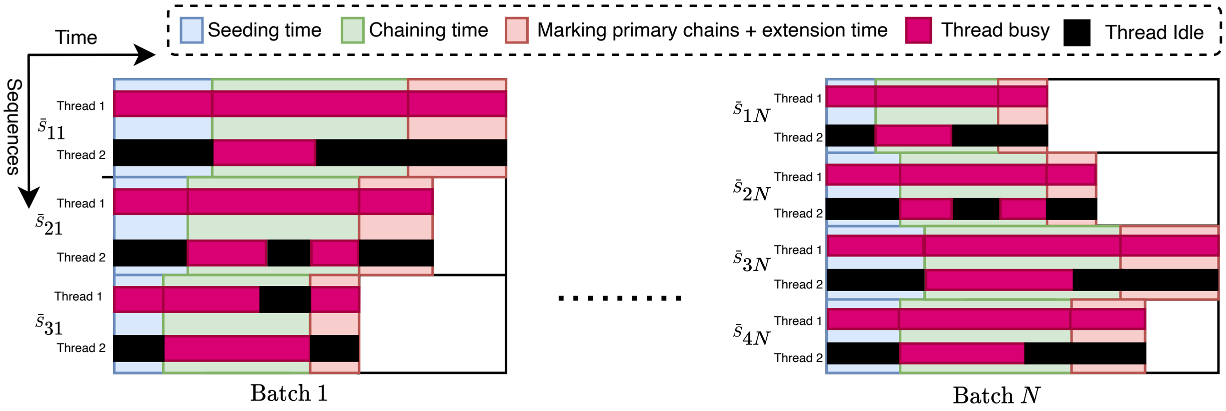

Figure S13: **Illustration of the overheads that affect CPU utilization of mm2-plus on a multi-core processor.** This schematic is intended for explanatory purposes only and does not represent actual experimental measurements. As described in the Methods section, mm2-plus and minimap2 process query genome sequences in batches. Let N denote the total number of batches. The finishing times of sequences within a batch are variable because sequence lengths are non-uniform. Assume that two threads are used per query sequence. The figure illustrates the overhead due to load imbalance within each batch (white blocks) and the overhead arising from sequential portions of the code (black blocks). The sequential portions exist in all stages of the pipeline, including seeding, chaining, marking primary chains, and extension.

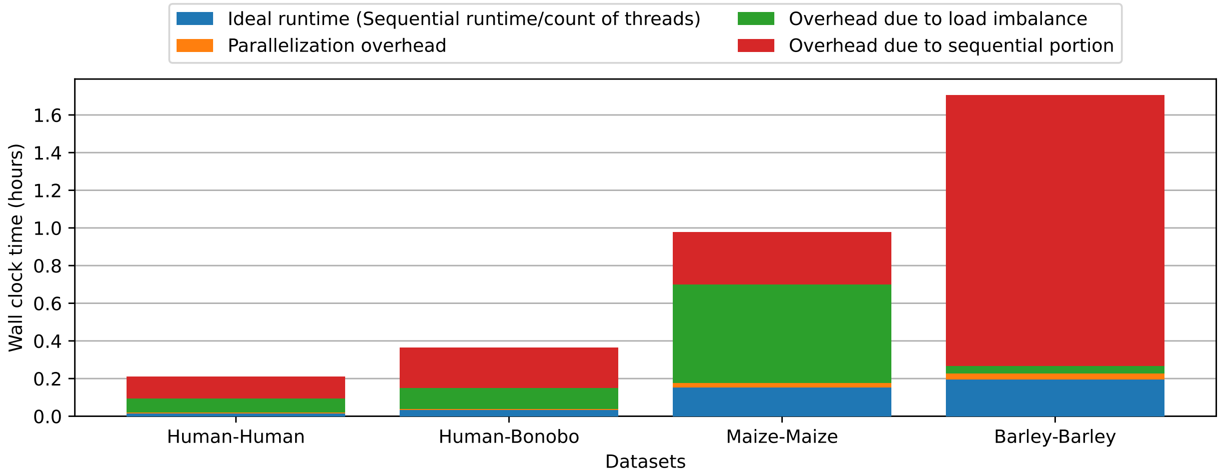

Figure S14: **Evaluation of various overheads in mm2-plus during parallel execution.** Fine-grained breakdown of overheads in mm2-plus across four datasets. We conducted this experiment using 48 threads of Cascade Lake CPU. Under ideal conditions with 100% parallel efficiency, the wall-clock time of mm2-plus would coincide with the ideal runtime shown in blue. We quantify the contributions of load imbalance, sequential code regions, and parallelization overheads to the observed slowdown. Across all datasets, a substantial fraction of the overhead arises from sequential portions of the code. The Barley–Barley dataset exhibits the largest sequential overhead, attributable to its higher N50 (Table S2A), which leads to multiple batches containing only a single query sequence. This overhead could be mitigated in future by developing fine-grained parallel algorithms for the seeding, chaining, and extension stages, or by reducing the memory-usage of minimap2 to enable larger batch sizes. Load imbalance overhead is also evident in three of the four datasets due to non-uniform query sequence lengths within batches.

Table S1: **Effect of increasing batch size on runtime and memory usage in minimap2 and mm2-plus.** "Batch size" refers to the total number of bases of the query genome processed in a single batch. The default batch size using in minimap2 (and mm2-plus) is 500 Mbp. We evaluated runtime and memory usage using two batch size parameters: 500 Mbp and 1000 Mbp. The results suggest that increasing batch size reduces runtime because fewer threads are idle but it also increases the memory usage. We used the default batch size (500 Mbp) in minimap2 and mm2-plus for all other experiments.

**Datasets**	**minimap2**				**mm2-plus**			
	**Batch size: 500M**		**1000M**		**Batch size: 500M**		**1000M**	
	Runtime (Hr)	Memory (GB)	Runtime (Hr)	Memory (GB)	Runtime (Hr)	Memory (GB)	Runtime (Hr)	Memory (GB)
Human–Human	0.33	29.61	0.23	43.01	0.20	31.25	0.16	43.12
Human–Bonobo	0.60	38.27	1.00	64.59	0.32	41.23	0.23	61.49
Maize–Maize	4.20	269.50	2.21	380.66	0.94	194.06	0.40	280.11
Barley–Barley	11.84	220.75	7.63	243.76	1.64	183.26	1.07	224.481

Table S2: **Details of the datasets used for benchmarking.**

(A) **Summary statistics of the genomes used for experiments**. We used genomes from *H. sapiens* (human), *P. paniscus* (bonobo), *Z. mays* (maize), and *H. vulgare* (barley) species.

**Genome**	**No. of sequences**	**Longest sequence (Mbp)**	**GC%**	**Genome length (Gbp)**	**N50 (Mbp)**
H. sapiens (CHM13 v2.0)	24	248.39	40.75	3.12	150.62
H. sapiens (HG002 PATERNAL)	23	252.06	40.68	2.95	146.79
P. paniscus (mPanPan1 PATERNAL)	31	227.70	40.37	3.24	147.03
Z. mays (Mo17 CAU 2.0)	10	307.34	46.92	2.18	220.30
Z. mays (W22 NRGENE 2.0)	191	310.93	45.86	2.13	222.59
H. vulgare (Morex V3)	290	665.59	44.45	4.23	610.33
H. vulgare (GPv1)	8	612.22	43.39	4.13	571.02

(B) For each dataset, we list the reference genome, query genome, and NCBI accession numbers below.

**Datasets**	**Reference**		**Query**	
	**Genome**	**Accession Number**	**Genome**	**Accession Number**
Human-Human	CHM13 v2.0 (Human)	GCA_009914755.4	HG002 PATERNAL (Human)	GCA_018852605.2
Human-Bonobo	CHM13 v2.0 (Human)	GCA_009914755.4	mPanPan1 PATERNAL (Bonobo)	GCF_029289425.2
Maize-Maize	Mo17 CAU 2.0 (Maize)	GCA_022117705.1	W22 NRGENE 2.0 (Maize)	GCA_001644905.2
Barley-Barley	Morex V3 (Barley)	GCA_904849725.1	GPv1 (Barley)	GCA_902500625.1

Table S3: **Commands used for running the experiments are listed in the table below**. Additionally, we provide automated scripts to reproduce the results in the mm2-plus GitHub repository (https://github.com/at-cg/mm2-plus/tree/v1.0/data/expts).

**Progressively enable optimizations in mm2-plus for benchmarking**	
baseline	`make base=1`
$+O_1$	`make avx=1`
$+O_2$	`make avx=1 opt_olp=1`
$+O_3$	`make avx=1 opt_olp=1 par_chain_1=1 par_btk=1`
$+O_4$	`make avx=1 opt_olp=1 par_chain_1=1 par_btk=1 par_sort=1`
**Whole-genome alignment using minimap2 and mm2-plus**	
Human-Human	`$EXECUTABLE -t48 -cx asm5 reference.fa query.fa > output.paf`
Human-Bonobo	`$EXECUTABLE -t48 -cx asm20 reference.fa query.fa > output.paf`
Maize-Maize	`$EXECUTABLE -t48 -cx asm5 reference.fa query.fa > output.paf`
Barley-Barley	`$EXECUTABLE -t48 -cx asm5 reference.fa query.fa > output.paf`
**Whole-genome alignment using mm2-gb**	
	`$EXECUTABLE -t1 -cx asm5 --gpu-chain --gpu-cfg gpu_config.json reference.fa query.fa > output.paf`
**Read alignment using minimap2, mm2-fast, and mm2-plus**	
HiFi	`$EXECUTABLE -t48 -ax map-hifi reference.fa reads.fq > output.paf`
ONT simplex/ultra-long	`$EXECUTABLE -t48 -ax map-ont reference.fa reads.fq > output.paf`
ONT duplex	`$EXECUTABLE -t48 -ax lr:hq reference.fa reads.fq > output.paf`
ONT all-vs-all	`$EXECUTABLE -t48 -x ava-ont reads.fq reads.fq > output.paf`
**Whole-genome alignment output quality evaluation by variant calling**	
PAF	`1) $EXECUTABLE -t48 --cs -cx asm5 reference.fa query.fa > minimap2.paf`
	`2) $EXECUTABLE -t48 --cs -cx asm5 reference.fa query.fa > mm2plus.paf`
PAF to VCF	`3) cat minimap2.paf | sort -k6,6 -k8,8n > minimap2_sorted.paf`
	`4) cat mm2plus.paf | sort -k6,6 -k8,8n > mm2plus_sorted.paf`
	`5) k8 paftools.js call -f reference.fa minimap2_sorted.paf | bgzip > minimap2.vcf.gz`
	`6) k8 paftools.js call -f reference.fa mm2plus_sorted.paf | bgzip > mm2plus.vcf.gz`
F1-score	`bcftools index --csi minimap2.vcf.gz`
	`bcftools index --csi mm2plus.vcf.gz`
	`bcftools isec -p out_dir -Oz minimap2.vcf.gz mm2plus.vcf.gz`
	`bcftools index --csi out_dir/0000.vcf.gz`
	`bcftools index --csi out_dir/0001.vcf.gz`
	`bcftools index --csi out_dir/0002.vcf.gz`
	`bcftools stats out_dir/0000.vcf.gz > out_dir/fn_stats.txt`
	`bcftools stats out_dir/0001.vcf.gz > out_dir/fp_stats.txt`
	`bcftools stats out_dir/0002.vcf.gz > out_dir/tp_stats.txt`
	`python3 get_f1.py`

Note: The script `get_f1.py` is available at https://github.com/at-cg/mm2-plus/blob/v1.0/data/expts/human/get_f1.py

Table S4: **Overview of the architectural details for the four processors used in the experiments: Emerald Rapids, Sapphire Rapids, Cascade Lake, and Milan.**

	Intel® Xeon®			AMD EPYC™
	**Platinum 8592+ (Emerald Rapids)**	**Platinum 8480L (Sapphire Rapids)**	**Platinum 6248R (Cascade Lake)**	**7763 (Milan)**
Sockets × Cores × Threads	1 × 64 × 2	1 × 56 × 2	1 × 24 × 2	1 × 64 × 2
AVX register width (bits)	512, 256, 128	512, 256, 128	512, 256, 128	256, 128
Vector Processing Units (VPU)	2/Core	2/Core	2/Core	2/Core
Base Clock Frequency (GHz)	1.90	2.30	3.00	2.45
L1D/L2 Cache (KB)	5120/131072	4480/114688	768/24576	2048/32768
L3 Cache (MB) / Socket	320	105	36.6	256
DRAM (GB) / Socket	256	256	357	251
Bandwidth (GB/s) / Socket	356	307	141	205
Compiler/Jemalloc/Zlib Version	GCC v13.2.0/ Jemalloc v5.3.0/ Zlib v1.2.11			

Table S5: **Comparison of variant calls obtained using mm2-plus and minimap2**. In this experiment, we considered the set of variants obtained using minimap2 alignments as ground truth. The high F1-scores obtained (last column) indicate a strong agreement between the alignments of both tools.

**Datasets**	**minimap2 Variants**				**Precision**	**Recall**	**F1-score**
	SNPs	Indels	SVs	Total			
Human-Human	2609943	600371	16082	3226396	0.999908	0.999898	0.999903
Human-Bonobo	35372859	3979201	102896	39454956	1.000000	1.000000	1.000000
Maize-Maize	2718820	237276	42517	2998613	0.999997	1.000000	0.999998
Barley-Barley	8882107	708383	320364	9910854	1.000000	1.000000	1.000000

Table S6: **Fraction of query and reference genome aligned using minimap2 and mm2-plus**. For each dataset, we calculated the fraction of bases in the reference genome and the fraction of bases in the query genome aligned by the methods. To get these values, we computed the size of the union of all alignment intervals on the query (reference) genome using Bedtools, and divided it by the size of the query (reference) genome.

**Datasets**	**minimap2**		**mm2-plus**	
	**Fraction (%)** (Reference genome)	**Fraction (%)** (Query genome)	**Fraction (%)** (Reference genome)	**Fraction (%)** (Query genome)
Human-Human	91.84	98.66	91.84	98.66
Human-Bonobo	91.51	91.60	91.51	91.60
Maize-Maize	61.62	65.39	61.62	65.40
Barley-Barley	88.57	90.10	88.58	90.12

Table S7: **Details of the long-read datasets used in our benchmark**. We used CHM13 T2T assembly (GCA_009914755.4) as the reference for mapping reads.

**Datasets**	**No. of reads**	**Longest read (kbp)**	**GC%**	**Coverage**	**N50 (kbp)**	**Accession No.**
PacBio HiFi	6,529,881	55	40.26	33.68×	16.99	SRR26402938
ONT simplex	2,356,641	1,694	40.83	12.65×	38.76	SRR24678051
ONT duplex	2,245,264	188	40.91	19.35×	35.97	SRR282957{59,61,65,66,71}
ONT ultra-long	1,139,047	966	40.54	16.55×	81.77	SRR24462105

Note: In our experiment, we down-sampled ONT ultra-long reads to 16.55× coverage.

Table S8: **Runtime and memory-usage comparison of mm2-gb and minimap2**. This table compares the runtime and memory usage of mm2-gb and minimap2. mm2-gb was evaluated on an NVIDIA A100-SXM4-40GB GPU paired with an AMD EPYC 7763 64-core CPU and 512 GB of RAM. Minimap2 was evaluated on a dual-socket Cascade Lake system using a total of 48 CPU threads. The longer runtime observed for mm2-gb is likely attributable to its limited parallelization: only the chaining stage is offloaded to the GPU, while the seeding and extension stages are executed on the CPU using a single thread. Indeed, the mm2-gb documentation states that "mm2-gb currently only supports single-threaded CPU execution (-t 1)." Configuration files required to reproduce these results are available in our GitHub repository (`https://github.com/at-cg/mm2-plus/blob/main/data/expts/gpu_config.json`). The commands used to run mm2-gb and minimap2 are listed in Supplementary Table S3.

**Datasets**	**mm2-gb**			**minimap2**	
	Runtime	Memory (CPU)	Memory (GPU)	Runtime	Memory (CPU)
Human–Human	0.60	50.3	35.7	0.33	29.61
Human–Bonobo	0.73	44.8	35.7	0.60	38.27
Maize–Maize	OOM	OOM	OOM	4.20	269.50
Barley–Barley	OOM	OOM	OOM	11.84	220.75

Note: "OOM" indicates that the experiment did not finish due to insufficient GPU memory.
